# Chemical Analysis and Biological Activity of the Essential Oils of Two Endemic Soqotri *Commiphora* Species 

**DOI:** 10.3390/molecules15020689

**Published:** 2010-02-01

**Authors:** Ramzi A. Mothana, Adnan J. Al-Rehaily, Wulf Schultze

**Affiliations:** 1 Department of Pharmacognosy, College of Pharmacy, King Saud University, P.O. Box 2457, Riyadh 11451, Saudi Arabia; E-Mail: ajalreha@ksu.edu.sa (A.J.A.); 2 Department of Pharmacognosy, Faculty of Pharmacy, Sana'a-University, P.O. Box 33039, Sana'a, Yemen; 3 Department of Pharmaceutical Biology and Microbiology, Institute of Pharmacy, Hamburg-University, Bundesstr. 45, D-20146 Hamburg, Germany; E-Mail: akschultze@uni-hamburg.de (W.S.)

**Keywords:** *Commiphora ornifolia*, *Commiphora parvifolia*, Soqotra, essential oil, antimicrobial, antioxidant, Yemen

## Abstract

The barks of two endemic *Commiphora *species namely, *Commiphora ornifolia* (Balf.f.) Gillett and *Commiphora parvifolia* Engl., were collected from Soqotra Island in Yemen and their essential oils were obtained by hydrodistillation. The chemical composition of both oils was investigated by GC and GC-MS. Moreover, the essential oils were evaluated for their antimicrobial activity against two Gram-positive bacteria, two Gram-negative bacteria and one yeast species by using a broth micro-dilution assay for minimum inhibitory concentrations (MIC) and for their antioxidant activity by measuring the DPPH radical scavenging activity. A total of 45 constituents of *C. ornifolia* (85.6%) and 44 constituents of *C. parvifolia* (87.1%) were identified. The oil of *C. ornifolia* was characterized by a high content of oxygenated monoterpenes (56.3%), of which camphor (27.3%), α-fenchol (15.5%), fenchone (4.4%) and borneol (2.9%) were identified as the main components. High contents of oxygenated sesquiterpenes (36.1%) and aliphatic acids (22.8%) were found in *C. parvifolia* oil, in which caryophyllene oxide (14.2%), β-eudesmol (7.7%), bulnesol (5.7%), T-cadinol (3.7%) and hexadecanoic acid (18.4%) predominated. The results of the antimicrobial assay showed that both oils exhibited moderate to high antibacterial activity especially against Gram-positive bacteria. *C. ornifolia* oil was the most active. In addition, the DPPH-radical scavenging assay exhibited only weak antioxidant activities for both oils at the high concentration tested.

## 1. Introduction

The genus *Commiphora* (Burseraceae) includes over 150 species of trees and shrubs, distributed mostly in East Africa, Arabia and India [1]. Besides the use of the resinous exudates of many *Commiphora* species in incense and perfumes, they redundant have been traditionally used for the treatment of sore stomach, colds, fever, malaria, wound healing, as an antiseptic and against skin infections [2,3,4]. Many *Commiphora* species were found to be of significant biological value for their cytotoxic, anti-inﬂammatory, antimicrobial, antimalarial, hypolipidemic, hepatoprotective and antioxidant effects [5,6,7,8,9,10,11,12,13,14,15,16]. 

The Soqotra Archipelago in Yemen has long been a land of mystery. Soqotra is considered the biodiversity "jewel" of the Arabian Sea. The long geological isolation of the Soqotra archipelago and its fierce heat and many droughts have combined to create a unique and spectacular endemic flora. Surveys have revealed that more than a third of the 800 or so plant species of Soqotra are found nowhere else [2]. The genus *Commiphora* is represented in Soqotra Island by five species: *C. kua* (Royle) Vollesen, *C. ornifolia* (Balf.f.) Gillett, *C. parvifolia* Engl., *C. planifrons* (Balf.f.) Engl. and *C. socotrana* (Balf.f.) Engl. [2]. The last four species are endemic to Soqotra. In the Soqotri folk medicine, *Commiphora* species are among the most important medicinal plants. The bark and underbark are used to make the most widely used powder dressing, called naqf. This is used on sores, ulcers and wounds of all sorts on both humans and livestock [2]. 

The present study was designed to analyze the chemical composition of the essential oils of two endemic *Commiphora* species, namely *C. ornifolia *and *C. parvifolia *as well as to evaluate their antimicrobial and antioxidant activities. 

## 2. Results and Discussion

### 2.1. Essential oil analysis

The volatile oils obtained after hydrodistillation of the barks of *C. ornifolia* and *C. parvifolia* gave an average yield of 0.31% and 0.19% on dry weight basis, respectively. The oil composition of the bark of both plants is presented in Table 1, in which the compounds are listed in order of their elution on the CP-Sil 5 CB column. In both oils more than 50 components were detected by GC/MS. Only 45 and 44 of them, representing about 85.6% of *C. ornifolia* and 87.1% of *C. parvifolia* respectively, could be identified by gas chromatographic and mass spectrometric data. It is important to mention that there have been no reports on GC-MS analysis of the essential oils of these two endemic *Commiphora* species. Additionally, this study represents the first report on the antimicrobial and antioxidant activities of both essential oils. Oxygenated monoterpenes were found as the major group of compounds in *C. ornifolia*, constituted 56.3% of the oil. Among them, camphor (27.3%) α-fenchol (15.5%), fenchone (4.4%) and borneol (2.9%) were identified as the main components. Caryophyllene oxide (6.5%) was the major compound among oxygenated sesquiterpenes (16.4%). *C. parvifolia* oil was characterized by high content of oxygenated sesquiterpenes (36.1%) and aliphatic acids comprised (22.8%). Among the oxygenated sesquiterpenes, caryophyllene oxide (14.2%), β-eudesmol (7.7%), bulnesol (5.7%) and T-cadinol (3.7%) were found to be the main constituents. Furthermore, hexadecanoic acid (18.4%) was determined as the major compound among the aliphatic acids. Oxygenated monoterpenes amounted only to 16.4% with camphor (9.1%) and α-fenchol (3.9%) dominating. The remaining unidentified components belong to various compound classes and their identification requires isolation and structure elucidation using 1D- and 2D-NMR spectroscopic methods. Our results appeared to be quite different from previously reported data on the chemical composition of *Commiphora* oils since they were devoid of monoterpene hydrocarbons. Such compounds e.g. α-pinene, β-pinene, *p*-cymene and α-thujene represented the most major compounds in many reported *Commiphora* oils [17,18,19,20]. Moreover, a previous study on the chemical composition of another Soqotri *Commiphora* species, namely *C. kua* [21], showed the absence of the major components in *C. ornifolia* and *C. parvifolia* such as camphor, α-fenchol, caryophyllene oxide and hexadecanoic acid. Generally, the comparison of our data with those of literature showed that the main constituents of chemical composition of the two investigated endemic *Commiphora* oils were markedly different from that of other known *Commiphora* species e.g., *C. myrrha, C. kataf, C. holtziana, C. shaerocarpa, C. kua* , *C. opobalsamum* and *C. africana* [17,18,19,20,21,22,23,24]. The data obtained can be used as a chemotaxonomical marker for *Commiphora* sp. from Soqotra. The difference of the composition of the oils could be attributed to the long geological isolation of geographical source as well as to the specific climate there.

### 2.2. Antimicrobial activity

The two essential oils were tested against two Gram-positive and two Gram-negative bacteria as well as against one fungal strain. The results of the bioassay exhibited that the oils had varying degrees of growth inhibition against the bacterial strains (Table 2). However, no activity was registered against *Candida albicans*. The Gram-negative strains showed less susceptibility to the tested essential oils than the Gram-positive ones. *C. ornifolia* essential oil demonstrated more activity (MIC values between 0.40 and 3.25 mg/mL) than that of *C. parvifoila* (MIC values between 2.18 and 8.75 mg/mL) (Table 2). This finding totally agrees with the observations derived from studies with essential oils from other *Commiphora* species [7,24]. Oxygenated monoterpenes such as camphor, borneol, linalool and α-terpineol, were reported to be responsible for the antimicrobial activity of several essential oils [25,26]. Consequently, the great antibacterial effect of *C. ornifolia* could be attributed to the high percentage of oxygenated monoterpenes such as camphor, α-fenchol, fenchon, borneol and α-terpineol. Possible synergistic effect of some compounds in the oils e.g. oxygenated sesquiterpenes (caryophyllene oxide, β-eudesmol, bulnesol and T-cadinol) as well as aliphatic acids e.g. hexadecanoic acid should also be taken in consideration. 

**Table 1:** Essential oil composition of *C. ornifolia* and *C. parvifolia*.

No.	Compounds	RI	*C. ornifolia*	*C. parvifolia*	Identification ^a^
1	Nonane	897	-	0.3	1,2
2	*t*-Linalool oxide	1059	0.2	-	1,2
3	Fenchone	1071	4.4	0.6	1,2,3
4	Linalool	1085	0.7	0.4	1,2,3
5	α-Fenchol	1104	15.5	3.9	1,2,3
6	*t*- Pinene hydrate	1110	1.8	0.3	1,2
7	Camphor	1127	27.3	9.1	1,2,3
8	Camphene hydrate	1138	0.3	-	1,2
9	Pinocarvone	1142	0.6	0.1	1,2
10	Borneol	1154	2.9	1	1,2,3
11	Terpinen-4-ol	1165	0.6	0.2	1,2,3
12	α-Terpineol	1175	1.2	0.4	1,2,3
13	Myrtenol	1182	0.7	0.3	1,2,3
14	Bornylacetate	1270	0.1	0.1	1,2
15	Theaspirane (Isomer I)	1295	-	0.7	1,2
16	α-Copaene	1379	0.1	0.1	1,2
17	(*E*)- β-Caryophyllene	1424	0.9	1.7	1,2,3
18	Gernaylacetone	1429	0.6	0.2	1,2
19	β-Farnesene	1450	0.5	-	1,2
20	α-Humulene	1457	0.3	0.7	1,2
21	β-Ionene	1467	0.2	-	1,2
22	γ-Muurolene	1476	0.5	0.3	1,2
23	β-Selinene	1488	0.4	0.4	1,2
24	α-Muurolene	1497	0.6	0.4	1,2
25	α-Alaskene	1512	0.3	-	1,2
26	δ-Cadinene	1517	0.5	0.5	1,2
27	Elemol	1540	-	0.5	1,2
28	*E*-Nerolidol	1549	0.8	0.1	1,2
29	*n*-Dodecanoic acid	1551	0.9	2.3	1,2
30	*t*- Dauca-4(11),7-diene	1557	0.4	0.9	1,2
31	Spathulenol	1574	1.1	1	1,2
32	Caryophyllene oxide	1580	6.5	14.2	1,2,3
33	α-Guaiol	1592	1.9	0.9	1,2
34	Humulene epoxide II	1604	0.9	1.9	1,2
35	γ-Eudesmol	1618	0.5	-	1,2
36	T-Cadinol	1632	1.4	3.7	1,2
37	β-Eudesmol	1645	1.6	7.7	1,2
38	α-Cadinol	1649	0.6	-	1,2
39	Bulnesol	1661	0.9	5.7	1,2
40	α-Bisabolol	1677	0.2	0.4	1,2
41	Pentadecanal	1693	0.4	0.5	1,2
42	*n*-Heptadecane	1702	0.2	-	1,2
43	*n*-Tetradecanoic acid	1745	-	1.8	1,2
44	Pentadecanoic acid	1842	-	0.3	1,2
45	Farnesylacetone	1894	-	0.3	1,2
46	Cemberene	1940	0.2	-	1,2
47	Hexadecanoic acid	1950	-	18.4	1,2
48	Cemberene A	1969	0.2	1.6	1,2
49	Thunbergol	2061	6.4	0.4	1,2
50	Manool	2078	2.7	0.5	1,2
51	Phytol	2114	-	5.8	1,2
52	Insensol	2195	3.8	1.1	1,2
	Oxygenated monoterpenes		56.3	16.4	
	Sesquiterpene hydrocarbons		4.7	5	
	Oxygenated sesquiterpenes		16.4	36.1	
	Aliphatic acids		0.9	22.8	
	Other compounds		8.2	6.8	

RI, Kovats indices relative to C_6_-C_28_ n-alkanes on the CP-Sil 5 CB column, ^a^: 1 = Kovats retention index, 2 = mass spectrum, 3 = co-injection with authentic compound.

### 2.3. Antioxidant activity

The potential antioxidant activity of the oils was determined on the basis of DPPH free radical scavenging activity. The investigated *Commiphora* oils demonstrated weak antioxidant abilities as estimated by their capability to reduce DPPH (Table 2). This observed effect could be associated with low content of phenolic components such as thymol and carvacrol in the two investigated *Commiphora* oils [27]. 

**Table 2:** Antimicrobial activity (MIC-values) and free radical scavenging activity of the investigated essential oils.

Plant species/reference	Radical scavenging activity in %					MIC ^a^				
	10	50	100	500	1000	*S. aureus*	*B. subtilis*	*E. coli*	*P. aeuginosa*	*C. albicans*
*C. ornifolia*	1.2	3.4	3.3	6.7	4.8	0.81	0.4	3.25	3.25	-
*C. parvifolia*	0.1	1.5	6.2	9.1	13.4	2.18	2.18	8.75	8.75	-
Amoxicillin						3.5	3.5	nt	nt	nt
Gentamicin						nt	nt	3.5	7	nt
Nystatin						nt	nt	nt	nt	3.5
Ascorbic acid	48.2	89.5	95.8	96.1	96					

^a^ minimum inhibitory concentration values are given as mg/mL for essential oils and μg/mL for standard antibiotics.

## 3. Experimental Section

### 3.1. Plant materials

The plants were collected from the Soqotra Island in the Winter of 2006 and identified at the Pharmacognosy Department, Faculty of Pharmacy, Sana'a University. Voucher specimens were deposited at the Pharmacognosy Department, Faculty of Pharmacy, Sana'a University. 

### 3.2. Isolation of the volatile oils

The air-dried and ground barks of *C*. *ornifolia* and *C*. *parvifolia* were submitted for 3 h to water distillation in a Clevenger-type apparatus. *n*-Heptane (1.5 mL) was used as a collector solvent. The obtained oils were dried over anhydrous sodium sulphate and after filtration and evaporation under nitrogen-flow, stored in sealed vials at +4 °C until tested and analyzed.

### 3.3. Gas chromatography analysis

The volatile oils were analyzed using a Hewlett Packard 5890 series II GC equipped with a Flame Ionization Detector (FID). The analysis was carried out on a CP-Sil 5 CB fused silica capillary column (Varian, 30 m × 0.25 mm i.d., film thickness 0.25 µm). Nitrogen was used as a carrier gas at a flow rate of 0.46 mL/min. Injector and detector temperature were set at 200 °C and 280 °C, respectively. Oven temperature was kept at 45 °C then gradually raised to 280 °C at 3 °C/min and finally held isothermally for 22 min. Diluted samples (1 μL, 1/100 in heptane, v/v) were injected manually (split mode, split ratio 1:16). Calculation of peak area percentage was performed on basis of the FID signal using the GC HP-Chemstation software (Agilent Technologies).

### 3.4. Gas chromatography-Mass spectrometry

The GC–MS analyses of the volatile oils were carried out using a Hewlett-Packard 5890 gas chromatograph coupled to a VG Analytical 70-250S mass spectrometer. The GC was equipped with a fused silica capillary CP-Sil 5 CB column (25 m × 0.25 mm i.d., ﬁlm thickness 0.40 µm, from Chromback, Varian). Helium was used as carrier gas at flow rate of 1 mL/min. The oven program started with an initial temperature of 80 °C held for 2 min and then the oven temperature was heated at 10 °C/min to 270 °C and finally held isothermally for 20 min. For GC-MS detection, an election ionization system, with ionization energy of 70 eV was used. A scan rate of 0.6 sec (cycle time: 0.2 sec) was applied, covering a mass range from 35 to 600 amu. 

### 3.5. Identification of components

The identification of the compounds was based on the comparison of retention indices and mass spectra of most of the compounds with data generated under identical experimental conditions by applying a two-dimensional searchalgorithm, considering the retention index as well as mass spectral similarity [28,29] or with those of authentic compounds available in our laboratories. Moreover, special software, namely the MassLib software (V9.3-106; 1996-2008, Max-Planck-Institute for Kohlenforschung, Muelheim, Germany) was used for processing and interpretation of mass spectra by comparison with the data from several commercially available libraries, including Wiley Registry of Mass Spectral Data (4^th^ Ed.), NIST/EPA/NIH Mass Spectral Library (2005), Library MPI Mühlheim (2006), Geochemicals (1^st^ Ed.), MRC collection (1^st^ Ed.). The retention indices (RI) were in relation to a homologous series of *n*-alkanes (C_6_– C_28_) on the CP-Sil 5 CB column under the same chromatographic conditions. Components relative concentrations were obtained by peak area normalization. No response factors were calculated.

### 3.6. Determination of antimicrobial activity

#### 3.6.1. Test organisms

The following microorganisms were used as test organisms in the screening: *Staphylococcus aureus* (BNI 18), *Bacillus subtilis* (BNI 28), *Escherichia coli* (BNI 2), *Pseudomonas aeruginosa *(BNI 20) and *Candida albicans* (BNI 33). The microbial strains were obtained from the Bernhard-Nocht-Institute (BNI) for Tropical Medicine, Hamburg, Germany.

#### 3.6.2. Broth micro-dilution assay for minimum inhibitory concentrations (MIC)

The broth micro-dilution method described by Mann and Markham [30] with modifications was used to determine the MIC of the investigated essential oils against the above mentioned microbial strains. With sterile round-bottom 96-well plates, duplicate two-fold serial dilutions of oil (100 µL/well) were prepared in the appropriate broth containing 5% (v/v) DMSO. Two-fold dilutions of amoxicillin, gentamicin or nystatin were used as a positive control. A bacterial cell suspension (prepared in the appropriate broth) of 100 µL, corresponding to 1 × 10^6^ CFU/mL, was added in all wells except those in columns 10, 11 and 12, which served as saline, essential oil and media sterility controls, respectively. Controls for bacterial growth without essential oil were also included on each plate. The final concentration of bacteria in the assay was 5 × 10^5^ CFU/mL. Plates were then incubated at 37 °C for 18 h overnight. After incubation, the MIC of each essential oil was determined as the lowest concentration at which no growth was observed in the duplicate wells. Twenty microliters of a *p*-iodonitro-tetrazolium violet solution (0.04%, w/v) (Sigma, USA) was then added to each well. The plates were incubated for a further 30 min, and estimated visually for any change in color from yellow to pink indicating reduction of the dye due to bacterial growth. The highest dilution (lowest concentration) that remained yellow corresponded to the MIC. Experiments were performed in duplicate.

### 3.7. Determination of antioxidant activity (scavenging activity of DPPH radical)

The DPPH free radical scavenging assay was carried out for the evaluation of the antioxidant activity. This assay measures the free radical scavenging capacity of the investigated oils. DPPH is a molecule containing a stable free radical. In the presence of an antioxidant which can donate an electron to DPPH, the purple color, typical for free DPPH radical decays, and the change in absorbency at λ = 517 nm is followed spectrophotometrically. This test provides information on the ability of a compound to donate a hydrogen atom, on the number of electrons a given molecule can donate, and on the mechanism of antioxidant action. The method was carried out as described previously [31]. The essential oils were dissolved in methanol and various concentrations (10, 50, 100, 500 and 1,000 µg/mL) of each oil were used. The assay mixture (total volume of 1 mL) contained 500 µL of the oil, 125 µL prepared DPPH (1 mM in methanol) and 375 µL solvent (methanol). After 30 min incubation at 25 °C, the decrease in absorbance was measured at λ = 517 nm. The radical scavenging activity was calculated from the equation: 

% of radical scavenging activity = Abs_control_ - Abs_sample_/ Abs_control_ × 100

## 4. Conclusions

In conclusion, the comparison of our results with those of literature showed that the main constituents of chemical composition of oils of the two investigated endemic *Commiphora *trees were markedly different from that of other known *Commiphora* species. The chemical profile of both oils could therefore be used as a chemotaxonomical marker to distinguish between the myrrh-trees on Soqotra Island and other places. Moreover, the results in the present study are in agreement with the traditional uses of the *Commiphora *trees investigated. Our results further support the idea that *Commiphora* sp. can be promising sources of potential antimicrobial agents. 
